# Correction: A review on fluorescent inorganic nanoparticles for optical sensing applications

**DOI:** 10.1039/c9ra90040c

**Published:** 2019-05-28

**Authors:** Andrew Shore

**Affiliations:** Royal Society of Chemistry Thomas Graham House, Science Park, Milton Road Cambridge UK advances-rsc@rsc.org

## Abstract

Correction for ‘A review on fluorescent inorganic nanoparticles for optical sensing applications’ by Sing Muk Ng *et al.*, *RSC Adv.*, 2016, **6**, 21624–21661.

This correction is being published to alert readers that Fig. 9, 19 and 21 have been reproduced from a *Sensors and Actuators B: Chemical* article, which has now been retracted. The article was cited as ref. 16 in this RSC Advances paper. The retraction notice of the *Sensors and Actuators B: Chemical* article,^1^ states that “this article reports a TEM image of the MAA capped CdS QDs (Fig. 2) which shows clear signs of manipulation (copy-pasting of the particles)”. Fig. 2 of the *Sensors and Actuators B: Chemical* article was reproduced in this *RSC Advances* article as Fig. 9.

The Royal Society of Chemistry apologises for these errors and any consequent inconvenience to authors and readers.

## Supplementary Material
